# Nursing workforce competencies and job satisfaction: the role of technology integration, self-efficacy, social support, and prior experience

**DOI:** 10.1186/s12912-023-01474-8

**Published:** 2023-09-07

**Authors:** Mohammed Hamdan Alshammari, Atallah Alenezi

**Affiliations:** 1 Mental Health Nursing Department, College of Nursing, University of Ha’il, Ha’il City, KSA Saudi Arabia; 2 College of Applied Medical Science, Master of Nursing in Mental Health, BSN, Shaqra University, Aldwadmi-Shaqra Road, 15572 Shaqra City, Riyadh Province Saudi Arabia

**Keywords:** Training, Technology Integration, Nursing workforce competencies, Job satisfaction, Self-efficacy, Social Support, Prior experience

## Abstract

**Background:**

The nursing profession has significant importance in delivering high-quality healthcare services. Nursing practitioners who have essential competencies and who are satisfied with their job are vital in achieving optimum patient outcomes. Understanding the effects of technology integration on nurse workforce competencies and job satisfaction is crucial due to the fast progress of technology in healthcare settings. Furthermore, many elements, including self-efficacy, social support, and prior experience have been recognized as possible mediators or moderators within this association. The primary objective of this quantitative research was to examine the influence of nursing education and the integration of technology on the competencies and job satisfaction of nursing professionals. Additionally, this study aimed to explore the potential mediating and moderating effects of self-efficacy and social support in this relationship.

**Methods:**

This cross-sectional, quantitative study employed an online survey questionnaire with standardized scales to measure nursing workforce competencies, job satisfaction, self-efficacy, social support, and prior experience. It was completed by 210 registered nurses from various healthcare settings in the Kingdom of Saudi Arabia. Data were analyzed by descriptive statistics, Pearson correlation analysis, multiple regression analysis, and structural equation modeling performed with SPSS 23 and SmartPLS 3.0 software.

**Results:**

The study’s findings revealed that nursing workforce competencies and job satisfaction were significantly predicted by nursing training and technology integration. The relationship between nursing training and technology integration, as well as nursing workforce competencies and job satisfaction, was partially mediated by self-efficacy and social support. Furthermore, prior experience moderated the relationship between nursing education and technological integration, nursing workforce competencies, and job satisfaction.

**Conclusions:**

The study’s findings suggest that nursing training and technology integration can improve nursing workforce competencies and job satisfaction and that self-efficacy and social support play an important role in mediating this relationship. Furthermore, prior experience can have an impact on the efficacy of nursing training and technology integration programs for developing nursing workforce competencies. The study has several practical implications for nursing education, training, and professional development programs, as well as strategies used by healthcare organizations to improve nursing workforce competencies and job satisfaction. To maximize their impact on nursing workforce competencies and job satisfaction, this study recommends that nursing training and technology integration programs focus on enhancing self-efficacy and social support. Furthermore, the study emphasizes the significance of prior experience when designing and implementing nursing training and technology integration programs.

## Introduction

The nursing profession is an important part of the healthcare industry and plays an important part in ensuring the health and well-being of patients [1]. The process of incorporating technological tools, resources, and systems into existing operations, practices, and environments is known as technology integration [2]. It involves integrating technology with established methods and strategies to attain particular objectives and results. The purpose of technology integration is to maximize the potential of technology to enhance processes, collaboration, communication, and overall performance [3]. The incorporation of technology into nursing education and practice in recent years has had a significant impact on the nursing workforce’s overall competency levels as well as their level of job satisfaction. The combination of nursing training and technological advancements has been found to improve the level of job satisfaction among nurses, which in turn results in better healthcare outcomes for patients [4].

The nursing workforce’s competencies and their level of satisfaction in their jobs are both essential components of nursing practice necessary for delivering high-quality care to patients [5]. Job satisfaction refers to the level of contentment and fulfillment that is experienced by nurses in their jobs, while nursing workforce competencies refer to the knowledge, skills, and abilities required for nurses to effectively perform their duties [6]. Nursing workforce competencies are important because they ensure that nurses have the knowledge and abilities necessary to provide care that is safe, effective, and centered on the patient. It is absolutely necessary to provide competent nursing care in order to reduce healthcare costs, improve patient satisfaction, and prevent unfavorable patient outcomes. Additionally, competent nursing care makes a contribution to the overall quality of the delivery of healthcare as well as the safety of the patient [7].

The retention of nurses is essential to the upkeep of a steady nursing workforce and job satisfaction is one factor that contributes to their motivation to stay in their positions. Researchers have found a correlation between high levels of job satisfaction and low rates of employee turnover, improved patient outcomes, and increased productivity [8]. Nurses who report higher levels of job satisfaction are also more likely to participate in activities that contribute to their ongoing learning and professional development, which in turn increases their level of expertise and efficiency as providers of healthcare [4].

Despite the fact that a number of studies have investigated the effect that nursing training and the incorporation of technology has had on the nursing workforce’s competencies and job satisfaction [9–11], there is a deficiency in the body of research concerning the factors that influence the success of these relations. To be more specific, there is a dearth of research on the ways in which individual differences, such as personality traits, moderate the relationship between nursing training and technology integration and nursing workforce competencies and job satisfaction [12].

The moderating role of prior experience in the effectiveness of nursing training and technology integration interventions highlights the necessity to consider individual differences when designing and implementing these interventions [13]. However, additional research is required on other potential moderators, such as personality traits, that may have an impact on the efficacy of nursing education and training programs. It is essential to gain an understanding of the moderating factors that have an effect on the efficacy of nursing education and training programs in order to design nursing interventions that are both more effective and better tailored to meet the varied requirements of the nursing workforce [12] In addition, nursing educators and practitioners can gain a better understanding of the underlying mechanisms of the interventions’ effects on nursing workforce competencies and job satisfaction by locating potential mediators such as self-efficacy and social support. Self-efficacy and social support were chosen as mediating variables for “individual differences” because they play important roles in shaping individual behavior, attitudes, and results [14, 15].

Self-efficacy is an individual’s belief in their own ability to complete activities and accomplish desired results. It determines how people approach problems, persevere in the face of adversity, and recover from disappointments. Higher degrees of self-efficacy promotes motivation, confidence, and goal-directed behavior, whereas lower levels might hamper performance and personal growth [16]. Social support, on the other hand, refers to the assistance, encouragement, and resources supplied by people inside an individual’s social network [17]. It could come from family, friends, coworkers, or managers. Social support is critical in relieving stress, increasing well-being, and facilitating adaptation to new situations or obstacles. Strong social support networks give emotional support, guidance, and practical aid, positively influencing an individual’s beliefs, behaviors, and overall functioning [18].

The objectives of this study were:

- To investigate the impact of nursing training and technology integration on nursing workforce competencies.
- To explore the relationship between nursing training and technology integration and job satisfaction among the nursing workforce.
- To examine the mediating role of self-efficacy and social support in the relationship between nursing training and technology integration and nursing workforce competencies and job satisfaction.
- To determine the moderating role of prior experience in the effectiveness of nursing training and technology integration interventions on nursing workforce competencies and job satisfaction.

These objectives aimed to fill a gap in the existing literature and provide a comprehensive understanding of the impact of nursing training and technology integration on nursing workforce competencies and job satisfaction, as well as the mediating and moderating factors that may influence the efficacy of these interventions. In addition, these objectives sought to identify the factors that may influence the effectiveness of these interventions. The conclusions from this study have the potential to inform nursing educators and practitioners on how to design and implement more effective training programs that cater to the diverse needs of the nursing workforce and better prepare them for the future of healthcare.

This research makes a significant contribution to the existing body of literature on nursing education and training by delivering an in-depth understanding of the influence that nursing training and the integration of technology have had on the competencies of nursing workforces and on job satisfaction. This study provides insight into how nursing educators and practitioners can design and implement more effective training programs that cater to the diverse needs of the nursing workforce. This was accomplished by exploring the mediating and moderating factors that influence the effectiveness of these interventions.

## Literature review

### Training and nursing workforce competence

Evidence from an ever-expanding body of research suggests that training has a material and beneficial effect on the nursing workforce’s level of competence. Simulation-based training programs have been found to greatly enhance nursing competence [19]. According to the findings of the study, the simulation-based training program increased nursing competence in a number of areas, including clinical judgment, communication, and care that is focused on the patient. The authors arrived at the conclusion that training based on simulation has the potential to be an efficient way of improving the nursing workforce’s competency. Moreover, well-structured and supervised clinical training greatly improves nursing competency [20]. Effective preceptorship programs, mentorship, and constructive feedback all contribute to the successful development of nursing competence during clinical training. Targeted educational interventions have been found to improve nursing competence [13]. Active learning tactics such as case studies, role-playing, and interactive conversations aid in knowledge retention and practical application, ultimately leading to increased nursing competence. In a similar vein, a review found that training interventions have a beneficial influence on nursing competence [21]. According to the findings of the review, training interventions, such as simulation-based training, clinical training, and educational interventions, significantly enhanced nursing competence. The authors came to the conclusion that educational interventions are necessary in order to improve the nursing workforce’s overall competence and the results for patients. Thus, the first hypothesis for this study was:

*H1: Training has a significant and positive impact on nursing workforce competence.*

### Training and job satisfaction

Training appears to have a large and beneficial effect on job satisfaction, according to a growing amount of research that has been conducted on the topic. Training has been found to have a beneficial impact on job satisfaction [14]. According to the findings of the study, job satisfaction was significantly higher among workers who had received training compared to workers who had not received training. The authors arrived at the conclusion that training was an important component that can have a considerable influence on occupational fulfillment. In a similar vein, training was found to have a favorable influence on job satisfaction [22]. According to the findings of the study, levels of job satisfaction were much greater for workers who had received training as opposed to workers who had not received training. The authors came to the conclusion that training was an essential component that can have a major effect on occupational fulfillment. Thus, the second hypothesis was:

*H2: Training has a significant and positive impact on job satisfaction.*

### Technology integration and nursing workforce competence

Evidence from a growing corpus of research demonstrates that the incorporation of technology has an important and beneficial effect on the nursing workforce’s level of competence. Technology integration has been found to have a beneficial effect on nursing workforce competence [23]. According to the findings of the study, nurses who made use of technology to assist them in their work exhibited higher levels of competence than their counterparts who did not make use of technology. The authors came to the conclusion that the incorporation of technology is a necessary component that has the potential to greatly influence nursing workforce competence. In a similar vein, another study found a favorable impact of technology integration on nursing workforce competency [24]. They found that technology integration has a favorable impact on nursing workforce competence. According to the findings of the study, nurses who made use of technology to assist them in their work exhibited higher levels of competence than their counterparts who did not make use of technology. The authors arrived at the conclusion that the incorporation of technology was an essential component that has the potential to greatly influence nursing workforce competence. Therefore, our third hypothesis:

*H3: Technology integration has a significant and positive impact on nursing workforce competence.*

### Technology integration and job satisfaction

The incorporation of technology into the working environment is now widely acknowledged as a critical component in the process of elevating employee happiness with their jobs. Many studies have been conducted to study the effect that the integration of technology has on job satisfaction, and all of them have revealed favorable findings. According to the findings of an investigation on the relationship between technology integration and job happiness, technology integration has a beneficial effect on job satisfaction [25]. According to the findings of the study, workers who had access to various forms of technology at their places of employment reported higher levels of job satisfaction than their counterparts who did not have such access. The authors arrived at the conclusion that the incorporation of technology was an essential component that can have a substantial effect on occupational fulfillment. In a similar vein, another study found that technology integration has a favorable influence on job satisfaction [26]. According to the findings of the study, levels of job satisfaction were much greater among workers who utilized technology to assist their work compared to workers who did not utilize technology. The authors arrived at the conclusion that the integration of technology was an important component that can have a major impact on occupational satisfaction. Therefore, our fourth hypothesis is:

*H4: Technology integration has a significant and positive impact on job satisfaction.*

### Self-efficacy and nursing workforce competence

The concept of self-efficacy relates to an individual’s belief in their own capacity to successfully carry out a specific activity or task. Research has been conducted to study the relationship between self-efficacy and nursing workforce competency, and all has revealed favorable findings. Self-efficacy was shown to have a favorable impact on nursing workforce competence [27]. According to the findings of the study, nurses who rated themselves as having high levels of self-efficacy had greater levels of nursing workforce competence than their colleagues who rated themselves as having low levels of self-efficacy. The authors came to the conclusion that self-efficacy was a crucial component that can have a considerable impact on the competence of the nursing workforce. Similarly, another study found that self-efficacy had a favorable influence on nursing workforce competence [4]. Based on the results of the investigation, it was observed that nurses who self-assessed their levels of self-efficacy as high exhibited higher levels of nursing workforce competence in comparison to their peers who self-assessed their levels of self-efficacy as low. The researchers reached the determination that self-efficacy was a significant factor that can exert a substantial influence on the proficiency of the nursing labor force.

*H5: Self-efficacy has a significant and positive impact on nursing workforce competence.*

### Self-efficacy and job satisfaction

Self-efficacy is a factor that can influence job satisfaction in the workplace. Employees who have high levels of self-efficacy are more confident in their skills to successfully carry out the responsibilities associated with their jobs. Much research has been conducted to study the relationship between self-efficacy and job satisfaction, and all of them have reported favorable findings. Researchers found that self-efficacy has a beneficial effect on job satisfaction [28]. According to the findings of the study, workers who rated themselves as having high levels of self-efficacy reported greater levels of job satisfaction than those workers who rated themselves as having low levels of self-efficacy. The authors concluded that self-efficacy was important and can have a considerable effect on occupational contentment.

*H6: Self-efficacy has a significant and positive impact on job satisfaction.*

### Social support and nursing workforce competence

The help, assistance, and comfort that an individual receives from their social network are all examples of what is referred to as social support. Numerous studies have shown positive findings on the relationship between social support and nursing workforce competence. Social support was found to have a beneficial effect on nursing workforce competence [29]. Registered nurses who had high levels of social support reported better levels of nursing workforce competence compared to those who had low levels of social support [30]. The authors concluded that social support is an important aspect that can have a substantial impact on the competence of nursing personnel.

*H7: Social support has a significant and positive impact on nursing workforce competence.*

### Social support and job satisfaction

Social support has been found to have a favorable influence on job satisfaction [31]. According to the findings of the study, workers who received high levels of social support reported higher levels of happiness in their jobs compared to workers who received low levels of social support. The authors concluded that social support was an essential component that can have a considerable effect on occupational fulfillment.

*H8: Social support has a significant and positive impact on job satisfaction.*

### Self-efficacy as a mediator

Self-efficacy increases as a result of training, which in turn leads to an improvement in the overall competence of the nursing staff. A sense of self-efficacy acts as a partial mediator of the connection between schooling and nursing workforce competence [4]. The authors came to the conclusion that education raised levels of self-efficacy, which in turn improved the competence of the nursing staff. In addition, self-efficacy acts as a moderator in the connection that exists between training and performance [32]. According to Bandura’s theory, self-efficacy may be improved by training, which in turn boosts performance and raises job satisfaction. The author made the argument that one of the most important factors that determine motivation, conduct, and performance was one’s views regarding one’s level of self-efficacy. Many researchers have investigated the connection between the incorporation of technology, self-efficacy, and the level of nursing workforce competency. In one such study, the incorporation of technology positively improved the competency of nursing personnel [22]. The authors also discovered that a person’s sense of self-efficacy acted as a mediator between the relationship between technology integration and the competence of the nursing staff. The authors came to the conclusion that increased use of technology led to greater feelings of self-sufficiency, which in turn led to improvements in nursing workforce competency. Another study looked into the relationship between the adoption of new technologies, feelings of self-efficacy, and levels of job satisfaction [22]. According to the findings of the study, the incorporation of technology had a favorable influence on occupational satisfaction. The authors also discovered that a sense of self-efficacy acted as a mediator between the relationship between integrating technology and feeling satisfied with one’s work. The authors concluded that greater use of technology led to greater feelings of self-efficacy, which in turn led to increased job satisfaction.

*H9a: Self-efficacy mediates the relationship between training and nursing workforce competence.*

*H9b: Self-efficacy mediates the relationship between training and job satisfaction.*

*H9c: Self-efficacy mediates the relationship between technology integration and nursing workforce competence.*

*H9d: Self-efficacy mediates the relationship between technology integration and job satisfaction.*

### Social support as a mediator

An individual who is struggling may receive social support in the form of emotional, informational, or physical assistance from the people around them. The provision of social support can come from a variety of different sources, such as managers and coworkers, relatives and friends, and friends of friends. The significance of social support in the nursing profession has recently come to light because it has the potential to influence both the level of care that nurses deliver and the overall enjoyment they derive from their work [33]. Additionally, feedback, mentoring, and resources can be provided via social support, which is another way it can boost the efficiency of training programs. The connection between social support and nursing workforce competency has been the subject of investigation. It was discovered that social support was a mediator of the association between nursing training and nursing competence [34]. To improve employees’ knowledge, abilities, and overall competence, it is necessary to implement training and development programs. On the other hand, training by itself might not be enough to guarantee that people are happy in their jobs. The connection between getting trained and being happy in one’s career might be mediated by having social support.

The relationship between the incorporation of technology and the competence of the nursing staff is mediated by social support. For instance, a study came to the conclusion that the social support that nurses received from their coworkers and superiors had a substantial influence in boosting their ability to make use of electronic health records [35]. Similarly, it was found that social support from mentors mediated the association between technology integration and nursing workforce competency [36]. Their findings were published in the journal Nursing Research. By providing employees with the resources and direction they require in order to properly use technology, social support was able to help reduce the negative consequences that technology use can have. For instance, coworkers can provide pointers and advice on how to use a particular piece of software or tool, while supervisors can provide training and support to ensure that employees have the skills necessary to carry out the responsibilities associated with their jobs. In addition, social support can help employees have a feeling of community and belonging in their workplace, both of which can contribute to increased levels of job satisfaction. When workers have the sense that they are a part of a community that cares about them, they are more likely to have a good outlook on their occupations and to be driven to do well in their work.

*H10a: Social support mediates the relationship between training and nursing workforce competence.*

*H10b: Social support mediates the relationship between training and job satisfaction.*

*H10c: Social support mediates the relationship between technology integration and nursing workforce competence.*

*H10d: Social support mediates the relationship between technology integration and job satisfaction.*

### Prior experience as a moderator

Nurses who have worked for longer periods may have already gained some of the knowledge and abilities that were taught in training programs as a result of their previous employment. As a result, they may gain more from training programs than nurses with less experience. On the other hand, nurses with less experience may have more to gain from training programs than those with more experience because they may have had fewer opportunities to develop the requisite skills and knowledge from their previous work experiences. Training programs can have a beneficial effect on job satisfaction by giving employees the knowledge, skills, and confidence they need to properly fulfill their job obligations. This has been demonstrated by research to be the case. Yet, the effect that training has on employees’ levels of happiness with their jobs may differ based on the amount of prior experience that each worker possesses. According to the findings of several studies, the experience can help regulate the relationship between the incorporation of technology and the nursing workforce’s level of competence. For instance, it was discovered that the positive association between technology integration and nursing workforce competency was larger for nurses who had more prior experience in utilizing technology [37]. Similarly, another study discovered that the association between technology use and nursing workforce competency was stronger for nurses who had a greater amount of prior experience [38].

*H11a: Prior Experience moderates the relationship between training and nursing workforce competence.*

*H11b: Prior Experience moderates the relationship between training and job satisfaction.*

*H11c: Prior Experience moderates the relationship between technology integration and nursing workforce competence.*

*H11d: Prior Experience moderates the relationship between technology integration and job satisfaction.*

### Conceptual model

**Fig. 1:**
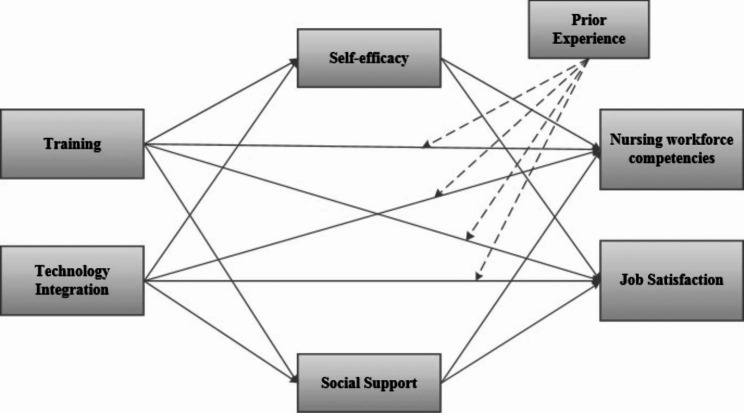
Conceptual Framework

The hypotheses from the literature review are connected in a conceptual model as shown in Fig. 1.

### Methods

#### Research design

In this cross-sectional, quantitative study, an online survey was used as the research method to investigate the impact of nursing training and technology integration on nursing workforce competencies and job satisfaction, as well as to investigate the moderating role of prior experience and the mediating roles of self-efficacy and social support.

### Setting

The population of registered nurses employed in healthcare settings in the Kingdom of Saudi Arabia were the focus of this study.

### Participants and sampling determination

The minimum desired sample size was determined using G-Power, applying the following parameters: a medium effect size of 0.30, a significance level of 0.05, and a power analysis of 0.95. By using these parameters, the minimum desired sample size for this study was 134. In order to aviod bias, a questionnaire was distributed to 300 targets using the convenience sampling method; 210 completed responses were received, exceeding the minimum desired sample size.

### Measures of the study varaibles

#### Training

A scale with three items that was taken from [39] and used to measure training. Items used to measure training were abbrivated T1, T2, and T3. Cronbach’s Aplha had a value of 0.776. Sample items included “My nursing training program adequately prepared me for the demands of the nursing profession”.

### Technology integration

A scale with three items that was taken from [40] and used to measure technology integration. Items used to measure technology intergration were abbrivated TI1, TI2, and TI3. The value of Cronbach’s Aplha was 0.822. Sample items included “Technology has made my job as a nurse easier and more efficient”.

### Nursing workforce competencies

A scale with three items was taken from [30] and used to measure nursing workforce competencies. Items used to measure workforece competencies were abbrivated NWC1, NWC2, and NWC3. The value of Cronbach’s Aplha was 0.888. Sample items included “I possess the necessary clinical skills to provide quality patient care.”

#### Job satisfaction

A scale with three items was taken from [41] and used to measure job satisfaction. Items used to measure job satisfaction were abbrivated JS1, JS2, and JS3. The value of Cronbach’s Aplha was 0.877. Sample items included “I find my work meaningful and fulfilling.”

### Self-efficacy

A scale with three items was taken from [42] and used to measure self-efficacy. Items used to measure self-efficacy were abbrivated SE1, SE2, and SE3. The value of Cronbach’s Aplha was 0.860. Sample items included “I am able to manage my time effectively to meet the demands of my job.”

#### Social support

A scale with three items was taken from [43] and used to measure social support. Items used to measure social support were abbrivated SS1, SS2, and SS3. The value of Cronbach’s Aplha was 0.823. Sample items included “I feel comfortable seeking help and support from my colleagues and supervisors.”

#### Prior experience

A scale with three items was taken from [44] and used to measure prior experience. Items used to measure prior experience were abbrivated PE1, PE2, and PE3. The value of Cronbach’s Aplha was 0.748. Sample items included “My prior experience has helped me to become a more effective nurse.”

#### Data collection

A platform for conducting online surveys, specifically Google Forms, was used to collect the data. The survey questionnaire consisted of four sections. In the first section, the participants were asked to provide basic demographic information, including age, gender, years of experience, and level of education. A validated instrument, the Nursing Competency Scale, was utilized in the second section in order to measure the nursing workforce’s level of competency [30]. The third section used a validated instrument, the Index of Work Satisfaction [41], to measure job satisfaction. In the fourth section, self-efficacy and social support were measured with instruments that have been previously validated [43, 44]. Data was collected from August 2, 2022 to November 20, 2022.

### Ethical considerations

Everyone who took part in the research project provided their informed consent, and participation in the study was entirely voluntary. Eliminating all personally identifying information from the questionnaires served to protect respondents’ privacy and maintain their anonymity.

### Data analysis

Data were analyzed by SPSS 21 and SmartPLS 3.0 software. Descriptive statistics were used to summarize the demographic characteristics of the sample. Pearson correlation coeffiecient was used to investigate the relationships between nursing training and technology integration, nursing workforce competencies, job satisfaction, self-efficacy, and social support. Analyses of mediation and moderation were carried out in order to investigate the roles that self-efficacy, social support, and prior experience play in mediating and moderating relationships through multiple resgression anlsysis using PLS-SEM software. Significance level was considered at p < 0.05.

## Results

### Sample profile

Out of the 210 surveys, 84% of the participants completed the questionnaire in Arabic, while 16% of the respondents did so in English. Table 1 lists the respondents’ demographic information. 42.9% of the responders were men and 57.1% were women based on gender. The bulk of respondents (28.6% and 27.1%, respectively), were in the age ranges of 30–40 years and 41–50 years. They represented a variety of experience, including 1 year (31%), 1–3 years (28.6%), 3–5 years (38.6%), and more than 5 years (1.9%).

**Table 1:** Demographic profile of the respondents

Demographic item		Frequency	Percentage
Gender	Male	90	42.9
	Female	120	57.1
Age	19–29	53	25.2
	30–40	60	28.6
	41–50	57	27.1
	51 years and above	40	19.0
Experience	1 year	65	31.0
	1–3 year	60	28.6
	3–5 years	81	38.6
	More than 5 years	4	1.8

### CFA loadings, reliability and validity test (convergent validity)

The concept indicators were compared to what was known about the variables’ nature using a confirmatory factor analysis (CFA). The CFA’s objective was to determine whether the data fit the suggested measurement model. The values of factor loading of all the items were greater than 0.4, which is shown in Table 2.

Furthermore, it was necessary to perform both a reliability test and a validity test on the data to investigate the validity and reliability of the collected data. This is an essential step in ensuring that empirical research maintains the highest standards of scientific integrity. Cronbach’s coefficient and combined reliability (CR) are two methods that are utilized in academic research to evaluate the reliability of a model. In addition, the model’s convergent validity was evaluated with the help of factor loadings and the average extracted variance (AVE). The findings are presented in Table 2, below. All of the variables’ indicators satisfied the requirements, which suggests that the variables chosen by the model have decisive reliability and internal consistency. The majority of academics use the criterion that the square root of AVE must be greater than the correlation coefficient between variables when evaluating the discriminant validity of a model. This is because this criterion ensures that the model accurately represents the world. As can be seen in Table 3, all values that are not on the diagonal have a lower absolute value than those values that are on the diagonal. This demonstrates that each variable in this model possessed good discriminative validity.

**Table 2:** CFA Loadings, Construct reliability and Validity (Convergent Validity)

	Items	CFA Loadings	Cronbach’s Alpha	CR	AVE
Job Satisfaction	JS1	0.913	0.877	0.924	0.803
	JS2	0.931			
	JS3	0.842			
Nursing Training	NT1	0.909	0.776	0.863	0.681
	NT2	0.882			
	NT3	0.663			
Nursing Work Competenceies	NWC1	0.881	0.888	0.931	0.818
	NWC2	0.919			
	NWC3	0.912			
Prior Experience	PE1	0.762	0.748	0.831	0.622
	PE2	0.726			
	PE3	0.871			
Self-Efficacy	SE1	0.836	0.860	0.902	0.754
	SE2	0.850			
	SE3	0.917			
Social Support	SS1	0.945	0.823	0.886	0.728
	SS2	0.622			
	SS3	0.951			
Technology Intergration	TI1	0.870	0.822	0.893	0.735
	TI2	0.875			
	TI3	0.827			

**Table 3:** Discriminant Validity (Fornell-Larcker)

	JS	NT	NWC	PE	SE	SS	TI
Job Satisfaction	0.896						
Nursing Training	0.086	0.825					
Nursing Work Competenceies	0.797	0.094	0.904				
Prior Experience	0.520	0.203	0.565	0.789			
Self-Efficacy	0.020	0.241	0.027	0.498	0.868		
Social Support	0.582	0.030	0.583	0.583	0.162	0.853	
Technology Intergration	0.607	0.397	0.468	0.101	0.280	0.414	0.857

JS = Job Satisfaction, NT = Nursing Training, NWC = Nursing Work Competenceies, PE = Prior Experience, SE = Self-Efficacy, SS = Social Support, TI = Technology Intergration

**Fig. 2:**
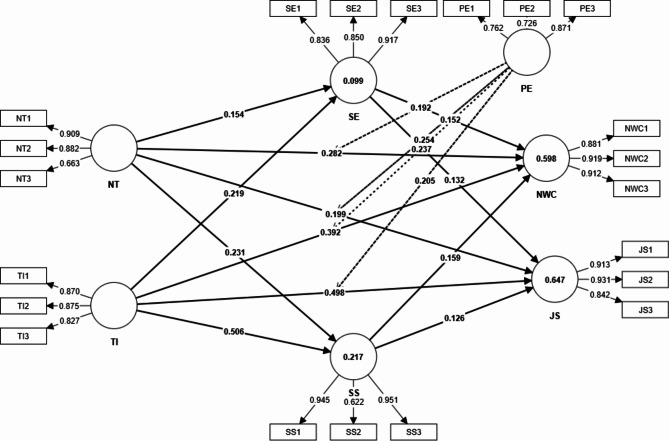
Measurement Model

#### PLS-SEM analysis

From the above reliability and validity analysis results, it is assumed that the model can carry out the next path impact analysis. Using SmartPLS software and the Bootstrapping method (n = 5000), the effect of each variable was estimated. All results were significant at the significance level of 0.5%, as shown in Table 4. Training and technology integration had a positive impact on nursing workforce competencies, with the path coefficients of 0.282 and 0.392, respectively, supporting hypotheses H1 and H3. Similarly, training and technology integration had a positive effect on job satisfaction with path coefficients of 0.199 and 0.498, respectively, supporting hypotheses H2 and H4. Moreover, self-efficacy and social support had positive impacts on nursing workforce competencies and job satisfaction with the path coefficients being 0.152 (H5), 0.159 (H7), 0.132 (H6) and 0.126 (H8), as shown in Fig. 2.

**Table 4:** Path Analysis

Constructs	Path coefficient	t-statistics	p-values
NT -> NWC	0.282	3.400	0.000
NT -> JS	0.199	2.671	0.004
TI -> NWC	0.392	6.842	0.000
TI -> JS	0.498	8.495	0.000
SE -> NWC	0.152	2.108	0.018
SE -> JS	0.132	2.100	0.018
SS -> NWC	0.159	2.792	0.003
SS -> JS	0.126	1.977	0.024

JS = Job Satisfaction, NT = Nursing Training, NWC = Nursing Work Competenceies, PE = Prior Experience, SE = Self-Efficacy, SS = Social Support, TI = Technology Intergration

#### Mediation analysis

Within the context of the relationship between news framing and public opinion, the purpose of this study was to investigate the mediating impact of self-efficacy between training and nursing workforce competence, training and job satisfaction, technology integration and nursing workforce competence, and technology integration and job satisfaction. The H9a hypothesis posited that self-efficacy mediates the relationship between training and nursing workforce competence. This hypothesis was supported by the findings, which show that self-efficacy mediates the relationship between training and nursing workforce competence (p = 0.000). In a similar vein, the H9b hypothesis posited that self-efficacy mediates the relationship between training and job satisfaction. This hypothesis was supported by the findings, which show that self-efficacy mediates the relationship between training and job satisfaction (p = 0.002). The H9c hypothesis posited that self-efficacy mediates the relationship between technology integration and nursing workforce competence. This hypothesis was supported by the findings, which show that self-efficacy mediates the relationship between technology integration and nursing workforce competence (p = 0.042). Similarly, The H9d hypothesis posited that self-efficacy mediates the relationship between technology integration and job satisfaction. This hypothesis was supported by the findings, which show that self-efficacy mediates the relationship between technology integration and job satisfaction (p = 0.048).

The H10a hypothesis posited that social support mediates the relationship between training and nursing workforce competence. This hypothesis was supported by the findings, which show that social support mediates the relationship between training and nursing workforce competence (p = 0.023). In a similar vein, the H10b hypothesis posited that social support mediates the relationship between training and job satisfaction. This hypothesis was supported by the findings, which show that social support mediates the relationship between training and job satisfaction (p = 0.005). The H10c hypothesis posited that social support mediates the relationship between technology integration and nursing workforce competence. This hypothesis was supported by the findings, which show that social support mediates the relationship between technology integration and nursing workforce competence (p = 0.005). Similarly, the H10d hypothesis posited that social support mediates the relationship between technology integration and job satisfaction. This hypothesis was supported by the findings, which show that social support mediates the relationship between technology integration and job satisfaction (p = 0.029). Table 5 shows the result of mediation analysis.

**Table 5:** Mediaiton Analysis

	Original Sample	t values	p values
NT -> SE -> NWC	0.023	5.175	0.000
NT -> SE -> JS	0.020	2.900	0.002
TI -> SE -> NWC	0.033	1.666	0.048
TI -> SE -> JS	0.029	1.728	0.042
NT -> SS -> NWC	0.037	2.002	0.023
NT -> SS -> JS	0.029	2.545	0.005
TI -> SS -> NWC	0.080	2.571	0.005
TI -> SS -> JS	0.064	1.894	0.029

JS = Job Satisfaction, NT = Nursing Training, NWC = Nursing Work Competenceies, PE = Prior Experience, SE = Self-Efficacy, SS = Social Support, TI = Technology Intergration

**Fig. 3:**
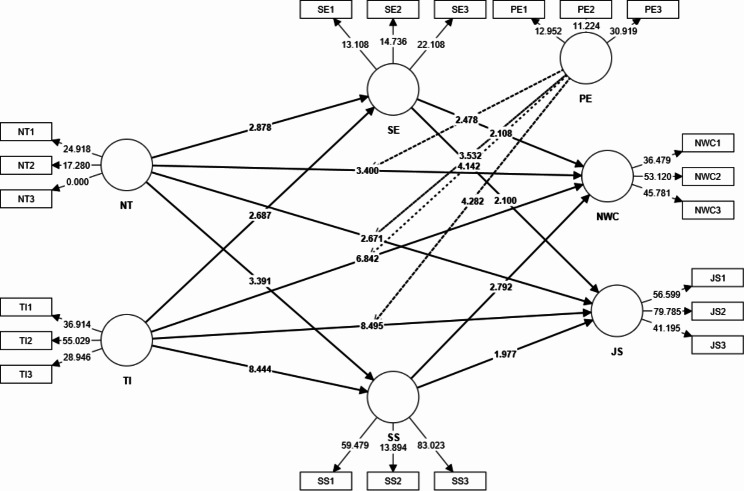
Structural Model

Hypotheses 11a,11b,11c, and 11d posited that prior experience moderates the relationship between training and nursing workforce competence, training and job satisfaction, technology integration and nursing workforce competence, and technology integration and job satisfaction. According to the findings of this study, prior experience acts as a moderating factor in the relationship between training and nursing workforce competence (p = 0.007), training and job satisfaction (p = 0.000), technology integration and nursing workforce competence (p = 0.000), and technology integration and job satisfaction(p = 0.000), confirming the hypotheses. The data collected during the study proves beyond a reasonable doubt the accuracy of these speculations. Table 6; Fig. 3 show the results of moderation analysis.

**Table 6:** Moderation Effect

	Original Sample	t values	p values
PE x NT -> NWC	0.192	2.478	0.007
PE x NT -> JS	0.254	3.532	0.000
PE x TI -> NWC	0.237	4.142	0.000
PE x TI -> JS	0.205	4.282	0.000

JS = Job Satisfaction, NT = Nursing Training, NWC = Nursing Work Competenceies, PE = Prior Experience, SE = Self-Efficacy, SS = Social Support, TI = Technology Intergration

## Discussion

The first hypothesis (H1) was supported with training having a significant and positive impact on nursing workforce competence. Nurses who participate in ongoing education are in a better position to handle difficult patient cases and are more conversant with the most recent treatment procedures and technological advances [19]. Training can also assist nurses in recognizing and responding appropriately to urgent circumstances, which can lead to improved results for their patients. The ability of nurses to engage with patients and their families is significantly improved when they receive training in communication skills. Increased patient satisfaction and decreased patient anxiety can be achieved through enhanced communication, which in turn leads to better clinical outcomes and a more positive patient experience [45].

The second hypothesis (H2) was supported with training having a significant and positive impact on job satisfaction. Training has a strong and favorable impact on both overall job satisfaction and individual job satisfaction. It enhances employee morale and motivation, creates possibilities for career growth, gives employees the skills and information necessary to properly fulfill their job obligations, and develops a culture of learning and development inside a business [14]. As a result, businesses ought to make investments in employee training and development programs in order to boost levels of job satisfaction and encourage the retention of employees. Training gives employees the skills and information they need to effectively do their jobs, leading to improved levels of job satisfaction overall. When workers believe they possess the relevant expertise to successfully carry out the responsibilities of their jobs, they experience an increase in self-assurance in their capabilities. This, in turn, contributes to a sense of accomplishment and overall job satisfaction [46].

The third hypothesis (H3) was supported with technology integration having a significant and positive impact on nursing workforce competence. Integration of new technologies has a materially beneficial effect on the professional level of the nursing workforce. It has improved communication and collaboration between healthcare professionals, improved access to patient data, provided advanced tools for diagnosis and treatment, improved the ability of healthcare professionals to communicate with one another, and made continuing education and training more accessible [47]. Healthcare organizations must maintain their investments in the incorporation of technology into nursing practice in order to enhance the nursing workforce’s level of competency and the outcomes for patients. Because of advancements in technology, nurses are now able to take part in continuing education and training programs, which has led to an increase in the overall competency of the nursing workforce. It is now much simpler for registered nurses to acquire training materials and continue their education while working thanks to the proliferation of online training programs, webinars, and other virtual learning platforms [48]. This has resulted in increased levels of job satisfaction among nurses, as well as improved levels of nursing skills and better outcomes for patients.

The fourth hypothesis (H4) was supported with technology integration having a significant and positive impact on job satisfaction. The implementation of technology has made it possible for employees to operate more effectively, collaboratively, and flexibly, which has contributed to increased levels of job satisfaction. According to the findings of one study, the incorporation of technology had a beneficial effect on occupational fulfillment, particularly in the areas of task diversity, autonomy, and feedback [49]. According to the findings of the study, employees were able to complete a greater diversity of jobs thanks to the implementation of technology in the workplace. This led to an increase in task variety, which was found to be positively associated with job satisfaction. Also, employees were able to work more independently and received more frequent feedback on their job performance as a result of technological advancements, which contributed to improved levels of job satisfaction.

The fifth hypothesis (H5) was supported with self-efficacy having a significant and positive impact on nursing workforce competence. Self-efficacy was found to have a favorable correlation with the problem-solving abilities of nurses, which are an essential component of nursing practice [4]. According to the findings of the study, nurses who had higher levels of self-efficacy were more confident in their ability to solve problems and were more competent when it came to managing difficult patient scenarios.

The sixth hypothesis (H6) was supported with self-efficacy having a significant and positive impact on job satisfaction. It has been discovered that an individual’s belief in their ability to perform a specific task or achieve a specific goal has a significant and positive impact on job satisfaction. Self-efficacy is defined as an individual’s belief in their ability to perform a specific task or achieve a specific goal. According to the findings of another study [17], a positive relationship exists between self-efficacy and job satisfaction among healthcare professionals. According to the findings of the study, healthcare professionals who had higher levels of self-efficacy were more engaged in their work and reported higher levels of job satisfaction than their counterparts who had lower levels of self-efficacy.

The seventh hypothesis (H7) was supported with social support having a significant and positive impact on nursing workforce competence. The supply of emotional, informational, or instrumental aid to a person by other people who are a part of that person’s social network is what is meant by the term “social support.“ Freshly graduated nurses who had social support reported higher levels of nursing competence and job satisfaction [18, 50]. According to the findings of the study, recently graduated nurses who received social support from their coworkers exhibited higher levels of nursing competence as well as greater levels of job satisfaction than those newly graduated nurses who did not receive social support.

The eighth hypothesis (H8) was supported with the impact of social support having a significant and positive impact on job satisfaction. It has been discovered that having a strong social support network has a large and favorable impact on the level of job satisfaction experienced by nurses. According to the findings of a study [51], a favorable association between social support and job satisfaction was identified among nurses. According to the findings of the study, nurses who reported receiving social support from their coworkers reported higher levels of job satisfaction than those nurses who did not report receiving social support.

Hypotheses H9a, H9b, H9c, and H9d investigated the mediating impact of self-efficacy between training and nursing workforce competence, training and job satisfaction, technology integration and nursing workforce competence, and technology integration and job satisfaction. All hypotheses were supported. Healthcare organizations place their primary emphasis on the development of training programs and the integration of technology in a manner that improves nurses’ sense of self-efficacy in order to boost both their level of professional achievement and their level of job satisfaction [14]. Students in the nursing profession who rated themselves highly in this area were more inclined to put into practice the skills and information they had acquired in school. Nurses’ job satisfaction can be boosted by training programs that boost their sense of competence. Mobile technology can help nurses feel more confident in their abilities, which increases the quality of care provided by the nursing profession as a whole. Self-efficacy in the use of electronic health records was associated with increased job satisfaction among nurses [52].

Hypotheses H10a, H10b, H10c, and H10d investigated the mediating impact of social support on the relationship between training and nursing workforce competence, training and job satisfaction, technology integration and nursing workforce competence, and technology integration and job satisfaction. All hypotheses were supported. In order to increase nurses’ level of expertise and overall level of job satisfaction, healthcare companies place a primary emphasis on cultivating a friendly working environment and fostering social support from coworkers and managers [21]. Nurses who had the backing of their superiors and peers throughout training were more likely to put what they had learned into practice, ultimately leading to a more capable nursing workforce. Nurses’ job happiness might be boosted by social support from coworkers and managers during training. Nurses’ ability to effectively implement clinical decision support systems was found to increase when they received social support from peers and superiors. This shows that a good outlook on work and increased job satisfaction might result from receiving social support from coworkers and superiors while using electronic health records.

Hypotheses H11a, H11b, H11c, and H11d investigated the impact of prior experience moderating the relationship between training and nursing workforce competence, training and job satisfaction, technology integration and nursing workforce competence, and technology integration and job satisfaction. All hypotheses were supported. To optimize the effects of training programs and the introduction of new technology on the competence and job satisfaction of the nursing workforce, healthcare organizations should take into account nurses’ varying degrees of experience [52]. Due to their presumably lower levels of expertise, nurses with less experience benefited more from training. Nurses with less experience may benefit more from training, and those who receive it may be more content with their careers. More seasoned nurses have a simpler time learning to use EHRs and other cutting-edge tools, which could boost the overall quality of the nursing staff [53]. In certain cases, nurses’ job satisfaction rises when they gain experience because they become more adept at using telehealth tools.

## Implications

Self-efficacy and social support are two crucial factors that play a mediating role in the relationship between nursing education and the incorporation of new technologies, on the one hand, and the competencies of the nursing workforce, on the other. The effect of nursing training and the incorporation of technology on nursing workforce competencies and job satisfaction is moderated, in part, by prior experience, which plays an important role in the process. The use of technology in nursing education has the potential to boost the self-efficacy of nurses, which in turn can have a positive impact on the level of job satisfaction and competency they experience. The effectiveness of nursing training and the incorporation of technology can be significantly improved with the addition of social support from coworkers and superiors. This can ultimately lead to an increase in nursing workforce competencies and job satisfaction. From a purely pragmatic standpoint, nursing organizations ought to put money into technological advancements that will improve nursing education and work on formulating plans for incorporating these advancements into the nursing workforce. The self-efficacy of nurses should be a primary focus of nursing education programs, along with the development of social support networks among coworkers and superiors. When developing nursing education programs and integrating technology, it is important to take previous experience into account. The health and happiness of employees should be a top priority for nursing organizations, and this can be accomplished by providing sufficient resources, support, and opportunities for training. The best way for nursing managers to improve their employees’ skills and overall job satisfaction is to create a supportive working environment that promotes teamwork and open communication among nursing staff members. It is possible for nursing organizations to improve the efficacy of their training programs and encourage a nursing workforce that is both more satisfied and more competent by taking into consideration the theoretical and practical implications discussed here.

### Limitations and future directions

Some limitations exist for the current study. In the first place, the research was only conducted in a single geographical area, which makes it difficult to extrapolate the findings to other countries or regions. The second limitation of the study was that it uses a cross-sectional methodology, which means that it is not possible to determine whether or not there is a cause-and-effect relationship between the different variables that were investigated. Self-reported measures of job satisfaction and competencies may also be subject to bias or social desirability effects, both of which have the potential to affect the accuracy of the results. Last, but not least, the research did not take into account any of the other potential moderating variables, such as age, gender, or educational background, all of which have the potential to affect the success of nursing training and technology integration.

To address these limitations, future research could use longitudinal designs to investigate the causal relationships between nursing training, technology integration, self-efficacy, social support, competencies, and job satisfaction. Furthermore, future research could look into the role of other potential mediators in the relationship between nursing training, technology integration, competencies, and job satisfaction, such as motivation, job demands, and work-life balance. Comparative research across regions or countries could also aid in identifying cultural, social, and organizational factors that influence the effectiveness of nursing education and technology integration. Furthermore, research could concentrate on developing and evaluating training interventions that promote self-efficacy and social support among nurses in order to improve their competencies and job satisfaction. Finally, research into the impact of technology integration on nursing training, workforce competencies, and job satisfaction is still in its early stages, and future studies could look into the long-term effects of technology integration on the nursing workforce. Researchers can improve our understanding of the impact of nursing training and technology integration on nursing workforce competencies and job satisfaction by addressing these limitations and pursuing these future directions.

## Conclusion

The current study emphasizes the significance of nursing education and technological integration in improving nursing workforce competencies and job satisfaction. Self-efficacy and social support were discovered to be important mediators of this relationship, with prior experience serving as a critical moderator. The incorporation of technology into nursing education can increase nurses’ self-efficacy, leading to increased job satisfaction and competencies. Social support from colleagues and superiors can significantly improve the effectiveness of nursing training and technology integration, promoting nursing workforce competencies and job satisfaction. Practical implications include investing in technology and developing strategies for its integration into the nursing workforce, prioritizing the well-being and job satisfaction of nursing staff, and fostering a supportive work environment. The study’s limitations include its cross-sectional design, self-reported measures, and regional focus. Future research could use longitudinal designs, investigate additional potential mediators and moderators, and investigate the long-term effects of technology integration on the nursing workforce. Overall, the current study emphasizes the significance of nursing education and technology integration in fostering a competent and satisfied nursing workforce.

## Data Availability

The datasets used and/or analyzed during the current study are available from the corresponding author on reasonable request.
